# Differentiating Multisystem Inflammatory Syndrome in Children (MIS-C) from Acute COVID-19 Using Biomarkers: Toward a Practical Clinical Scoring Model

**DOI:** 10.3390/biomedicines14020258

**Published:** 2026-01-23

**Authors:** Carmen Loredana Petrea (Cliveți), Diana-Andreea Ciortea, Gabriela Gurău, Mădălina Nicoleta Matei, Alina Plesea Condratovici, Andreea Eliza Zaharia, Codrina Barbu (Ivașcu), Gabriela Isabela Verga (Răuță), Sorin Ion Berbece

**Affiliations:** 1 Faculty of Medicine and Pharmacy, Research Center in the Medico-Pharmaceutical Field, “Dunarea de Jos” University of Galati, 800008 Galati, Romania; carmen.petrea@ugal.ro (C.L.P.); madalina.matei@ugal.ro (M.N.M.); alina.plesea@ugal.ro (A.P.C.); andreea.zaharia@ugal.ro (A.E.Z.); codrina.barbu@ugal.ro (C.B.); gabriela.verga@ugal.ro (G.I.V.); sorin.berbece@ugal.ro (S.I.B.); 2 Sf. Ioan’ Emergency Clinical Hospital for Children, 800487 Galati, Romania; 3 “Maria Sklodowska Curie” Emergency Clinical Hospital for Children, 041451 Bucharest, Romania

**Keywords:** MIS-C, COVID-19 pediatric, inflammatory biomarkers, NLR, PLR, electrolyte imbalance, machine learning

## Abstract

**Background/Objectives**: Severe acute respiratory syndrome coronavirus 2 (SARS-CoV-2) infection in children presents with a heterogeneous clinical spectrum, whereas multisystem inflammatory syndrome in children (MIS-C) is a distinct immunological entity characterized by a hyperinflammatory phenotype and a distinct biological architecture. Identifying routine biomarkers with early discriminatory utility is essential for rapid differentiation between MIS-C and coronavirus disease 2019 (COVID-19). **Methods**: We conducted a retrospective comparative study of 144 pediatric patients with COVID-19 or MIS-C admitted to a single specialized medical center. The analyses integrated classical statistical methods, Benjamini–Hochberg false discovery rate correction (FDR), penalized regression models, and machine learning algorithms to identify biomarkers with discriminative value, using only routine laboratory tests. **Results**: MIS-C was associated with an intense inflammatory profile, characterized by increases in C-reactive protein (CRP), neutrophil-to-lymphocyte ratio (NLR), and platelet-to-lymphocyte ratio (PLR), lymphopenia, and selective electrolyte disturbances, highlighting a coherent biological architecture. In contrast, COVID-19 showed limited associations with traditional inflammatory markers. Predictive models identified a stable core of biomarkers with excellent performance in Random Forest analysis (area under the curve, AUC = 0.95), and reproducible thresholds (CRP ~3.7 mg/dL, NLR ~3.3, PLR ~376; potassium ~4.2 mmol/L). These findings were independently confirmed using penalized Ridge regression, where the reduced model achieved superior discrimination compared to the full 13-variable model (AUC = 0.93 vs. 0.89) and maintained stable performance under internal cross-validation, reinforcing the clinical relevance of this compact biomarker panel. **Conclusions**: MIS-C is clearly distinguished from COVID-19 by a specific and reproducible immunological signature. The identified biomarkers may represent a potential foundation for the development of simple clinical algorithms for pediatric triage and risk stratification, opening the prospect of a simplified scoring tool applicable in emergency settings.

## 1. Introduction

Severe acute respiratory syndrome coronavirus 2 (SARS-CoV-2) infection and its post-viral complications, particularly multisystem inflammatory syndrome in children (MIS-C), have posed significant challenges in pediatrics, both in terms of differential diagnosis and clinical management [1,2,3]. The variability of manifestations—from asymptomatic coronavirus disease 2019 (COVID-19) infection to severe multisystem involvement—has highlighted the complexity of this disease in children, which is much more pronounced than initially anticipated [4,5].

Although SARS-CoV-2 infection generally causes mild or moderate clinical forms in the pediatric population [5], emerging data have highlighted substantial heterogeneity in immune responses, including severe and dysregulated inflammatory reactions. In children, COVID-19 most commonly presents with respiratory involvement; however, systemic disturbances may also occur through mechanisms that remain incompletely understood [6,7,8,9,10,11,12,13,14]. Beyond the acute viral phase, SARS-CoV-2 infection has been associated with delayed immune dysregulation and atypical post-infectious inflammatory responses. While MIS-C represents the most frequently described hyperinflammatory post-infectious entity in the pediatric population [6,7], increasing evidence suggests that SARS-CoV-2 infection may also act as trigger for a broader spectrum of immune-mediated conditions. New-onset autoimmune diseases, including connective tissue disorders and systemic autoimmune diseases such as systemic lupus erythematosus, have been reported following SARS-CoV-2 infection in both adult and pediatric populations [15,16,17,18,19,20]. These observations support the concept of post-infectious immune dysregulation after SARS-CoV-2 infection, with MIS-C representing the dominant pediatric phenotype, but not the sole immune-mediated manifestation.

MIS-C is a rare but potentially life-threatening post-infectious complication occurring in children and teenagers with recent or confirmed SARS-CoV-2 infection, typically manifesting 2–6 weeks after the acute phase [21,22]. Since its first description in 2020, several diagnostic frameworks have been proposed by international organizations, including the World Health Organization (WHO), the European Centre for Disease Prevention and Control (ECDC), and the US Centers for Disease Control and Prevention (CDC). All diagnostic frameworks emphasize a post-infectious onset, marked systemic inflammation, and the exclusion of alternative infectious causes [23,24,25]. The terminology varies between “Pediatric Inflammatory Multisystem Syndrome Temporally Associated with SARS-CoV-2” (PIMS-TS) and “Multisystem Inflammatory Syndrome in Children and Adolescents Temporally Related to COVID-19” (MIS-C). However, these terms describe the same clinical entity with a heterogeneous phenotypic spectrum [2,3]. In the present study, the MIS-C definition proposed by WHO was applied consistently.

From a pathogenic perspective, MIS-C is a distinct clinical entity, mediated by an aberrant immune response, with massive release of proinflammatory cytokines, including interleukins (IL-6, IL-1β, IL-12), activation of immunological markers such as LAMP-1, IFNGR2, CD244 proteins, and the formation of immune complexes involving immunoglobulin G (IgG), immunoglobulin A (IgA), and immunoglobulin M (IgM), as well as autoantibodies (anti-La and anti-aminoacyl t-RNA synthetase). These processes lead to systemic hyperinflammation, endothelial dysfunction, vasculitis, and multiorgan damage [23,26]. In contrast to acute COVID-19, MIS-C is more frequently associated with cardiovascular dysfunction, electrolyte disturbances, and marked hematological abnormalities, leading to increased disease severity and need for intensive care [21,27]. These overlapping yet distinct features complicate early differentiation from acute COVID-19, particularly when clinical and laboratory findings are shared [28,29].

In the absence of universal access to advanced immunological tests, the identification of routinely available biomarkers such as C-reactive protein (CRP), complete blood count, derived hematological ratios (neutrophils-to-lymphocytes, NLR; platelets-to-lymphocytes, PLR), serum sodium and potassium, and D-dimer levels, becomes essential for early discrimination between COVID-19 and MIS-C [30,31,32,33,34,35,36]. Despite extensive investigation of these markers, comparative studies translating them into simple, data-driven clinical models validated in pediatric practice remain limited. Consequently, standardized tools or widely applicable scores based exclusively on routine biomarkers are currently lacking, particularly in settings where access to advanced immunological techniques is restricted. Recent integrative analyses have additionally suggested that electrolyte disturbances and antidiuretic hormone (ADH)-mediated mechanisms may act as complementary components within the inflammatory biomarker landscape of MIS-C, supporting their potential inclusion in multidimensional biological profiling [37].

In this context, the present study aims to systematically compare the clinical and biological profiles of cases diagnosed with MIS-C and acute COVID-19 in a pediatric cohort, using only routine biomarkers. The main objective of this study is to identify routine biomarkers with discriminatory value and to evaluate the feasibility of constructing a simplified prediction model—such as a clinical score or decision tree—to support rapid triage and differential diagnosis. This approach is particularly relevant in pediatric patients who present with overlapping MIS-C and acute COVID-19 phenotypes, in which clinical manifestations and inflammatory markers may partially overlap, and diagnostic uncertainty is high.

## 2. Materials and Methods

### 2.1. Study Design

We conducted a retrospective observational single-center study of pediatric patients admitted to the “Sf. Ioan” Emergency Clinical Hospital for Children in Galati, Romania, over four years (1 July 2020–30 June 2024). The study included children diagnosed with acute COVID-19 infection or MIS-C.

### 2.2. Diagnosis and Clinical Classification

MIS-C was diagnosed according to internationally accepted case definitions issued by the CDC, subsequently updated, and endorsed by the WHO [28]. Acute COVID-19 infection was confirmed by the detection of SARS-CoV-2 ribonucleic acid (RNA) using real-time reverse transcription polymerase chain reaction (qRT-PCR). Disease severity was classified according to current international guidelines for pediatric COVID-19 and MIS-C [28,38].

### 2.3. Biological Determinations and Variables

All laboratory investigations were performed in the hospital’s own certified clinical laboratory using standardized procedures, and results were interpreted in accordance with age-specific pediatric reference ranges. 

SARS-CoV-2 RNA was detected by qRT-PCR after viral RNA extraction with the Seegene Nimbus system (Seegene Inc., Seoul, Republic of Korea) and amplification on the CFX96 platform (Bio-Rad Laboratories, Hercules, CA, USA). Serological detection of SARS-CoV-2 IgG and IgM antibodies was performed by chemiluminescence using the YHLO-IFLASH 1800 system (YHLO Biotech Co., Ltd., Shenzhen, China). Complete blood counts were processed on automated hematology analyzers (Mindray BC-6200 and Celltac G 9100/9200). Biochemical analyses, including serum electrolytes and bicarbonate (ECO_2_), were performed using the Vitros 4600 Chemistry System (Ortho Clinical Diagnostics, Rochester, NY, USA).

Inflammatory status was assessed using routinely available hematological and biochemical parameters, including complete blood count, CRP, fibrinogen, D-dimer, erythrocyte sedimentation rate (ESR), and serum electrolytes (sodium, potassium and bicarbonate). Bicarbonate was assessed as alkaline reserve (AR), expressed as estimated total CO_2_ (ECO_2_), according to the laboratory reporting system of study center. Two derived inflammatory indices were calculated from the complete blood count—NLR and the PLR—reflecting combined neutrophilic activation, lymphocyte depletion, and platelet involvement in systemic inflammation.

The selection of NLR and PLR indices was based on both pathophysiological and practical considerations. These indices are derived from routine blood counts, are universally available, can be obtained rapidly, and do not require specialized testing. In the context of MIS-C, NLR reflects the combined effect of neutrophilia and lymphopenia. PLR integrates platelet activation with systemic inflammation, two essential components of the hyperinflammatory phenotype described in this condition. Although numerous inflammatory biomarkers are available, NLR and PLR offer a robust, reproducible, and clinically accessible means of capturing immune dysregulation, making them particularly suitable for comparative analyses and the development of simplified predictive models.

### 2.4. Inclusion and Exclusion Criteria

The study population included pediatric patients aged 0–18 years diagnosed with either acute COVID-19 or MIS-C during the study period.

The MIS-C cases (*n* = 36) fulfilled the diagnostic criteria described above and were included consecutively.

Acute COVID-19 cases were identified among all children with SARS-CoV-2 infection confirmed by qRT-PCR and managed during the same period (*n* = 599). Considering the main objective of the study, which was to compare biological phenotypes between MIS-C and acute SARS-CoV-2 infection, a COVID-19 subgroup (*n* = 108) was selected using a 1:3 matching ratio with the MIS-C group (*n* = 36). Matching was performed strictly by age and sex, and controls were selected from consecutive hospitalizations to reduce selection bias and increase the validity of the comparison. Appropriate COVID-19 controls were identified for all MIS-C cases.

A minimum length of hospitalization (LOS) of 48 h was required to ensure consistent clinical and laboratory assessment. Given the retrospective and observational design, no a priori sample size calculation was performed.

Patients were excluded if relevant laboratory data were unavailable or if they had a history of significant comorbidities, including renal failure or renal transplantation (estimated glomerular filtration rate < 15 mL/min./1.73 m^2^ or dialysis-dependent), heart failure, or other organ failure. Pediatric patients receiving treatment for chronic conditions, as well as those whose MIS-C diagnosis was revised during or after hospital admission, were also excluded. Additionally, patients discharged within 48 h, managed on an outpatient basis, or transferred to dedicated COVID-19 units were excluded from subgroup selection.

### 2.5. Statistical Analysis

The statistical analyses were performed using SPSS v26 software (IBM Corp., Armonk, NY, USA) and Python (version 3.11.8) in a Jupyter Notebook (version 7.1.3) environment.

Continuous variables are summarized as means ± standard deviations for approximately normally distributed data or as medians with interquartile ranges for non-normally distributed variables. Categorical variables are expressed as frequencies and percentages.

Data distribution was evaluated descriptively, and the choice between parametric and nonparametric statistical tests was made accordingly. Group comparisons between MIS-C and COVID-19 were performed using the independent samples *t*-test for normally distributed variables or the Mann–Whitney U test for non-normally distributed data.

Categorical variables were compared using the Chi-square test or Fisher’s exact test, as appropriate. For comparisons involving multiple groups, the Kruskal–Wallis H test was applied, followed by post hoc analyses with Benjamini–Hochberg false discovery rate (FDR) correction when indicated.

Associations between continuous variables were assessed using Pearson correlation coefficients for approximately normally distributed data and Spearman rank correlation coefficients for non-normally distributed variables.

The ability of biological markers to discriminate between MIS-C and acute COVID-19 was explored using logistic regression and tree-based classification models, with performance evaluated by the area under the receiver operating characteristic curve (AUROC). Random Forest models were applied to assess multivariate classification patterns and relative variable importance. Additional methodological details regarding the machine-learning analyses are provided in the Supplementary Methods.

Pareto analysis was used exclusively as a descriptive visualization tool to illustrate percentage differences in the prevalence of biological abnormalities between the two cohorts. Its approach was intended to highlight the relative magnitude of group differences and was not used for feature selection or predictive modeling.

Selection of candidate biomarkers for multivariate and machine-learning analyses was based on a combination of clinical relevance, biological plausibility, and data availability. Only routinely available laboratory parameters with sufficient completeness across the study cohort were considered. Exploratory univariate comparisons and correlation analyses were additionally used to identify markers with potential discriminatory value between MIS-C and acute COVID-19. This stepwise selection strategy was implemented to ensure model robustness, interpretability, and clinical applicability, while minimizing the risk of overfitting. Statistical significance was set at a two-tailed *p*-value < 0.05.

### 2.6. Ethical Approval and Informed Consent

This retrospective study was approved by the Medical Council of the “Sf. Ioan” Children’s Emergency Hospital (approval no. C399/30.08.2024). The approval was subsequently updated on 7 November 2025 (document no. 24875) to extend institutional authorization for data use and publication, without any changes to the study design, patient population, or study procedures.

In accordance with institutional regulations, written informed consent is routinely obtained from parents or legal guardians at the time of hospital admission, prior to the performance of any medical procedures. This general informed consent includes permission for the use of anonymized clinical data for medical research purposes. Given the retrospective design of the study and the use of anonymized data, no additional informed consent was required for the present analysis.

## 3. Results

Comparative analyses were performed between 36 pediatric patients diagnosed with MIS-C and 108 pediatric patients with COVID-19, matched for age and sex, to identify clinical and biological differences between the two conditions.

### 3.1. Distribution of Clinical Forms and Length of Hospitalization (LOS)

**Figure 1:**
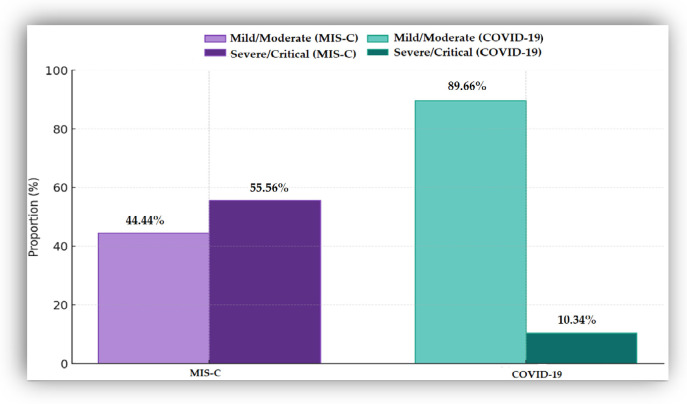
Distribution of clinical forms in the MIS-C and COVID-19 groups. The *x*-axis shows the two study cohorts, while the *y*-axis represents the proportion (%) of mild/moderate and severe/critical clinical forms. MIS-C (*n* = 36); COVID-19 (*n* = 86).

The distribution of clinical forms differed significantly between the two groups. In the COVID-19 cohort (*n* = 108), 21 patients (19.44%) were asymptomatic and were excluded from the severity analysis. After reclassification of the clinical forms as mild/moderate versus severe/critical, the proportion of severe/critical forms was higher in the MIS-C group (55.56%) than in the symptomatic COVID-19 group (10.34%) (Figure 1).

LOS was significantly longer in the MIS-C group, with a median of 10 days (IQR: 7–12) compared with 2 days (IQR: 2–5) in the COVID-19 group.

### 3.2. Comparative Biomarker Profiles Between MIS-C and COVID-19

To characterize the biological differences between MIS-C and COVID-19, the main inflammatory, hematological, and metabolic biomarkers were compared across the two groups.

Comparative analysis using the Mann–Whitney U test revealed significantly more pronounced inflammatory and coagulation marker profiles in the MIS-C group, accompanied by leukocytosis, neutrophilia, lymphopenia, and increased NLR and PLR values (Table 1). Serum sodium and potassium were significantly lower in MIS-C, while platelet counts did not differ significantly between groups (Table 1). AR showed no significant differences (*p* = 0.3398).

**Figure 2:**
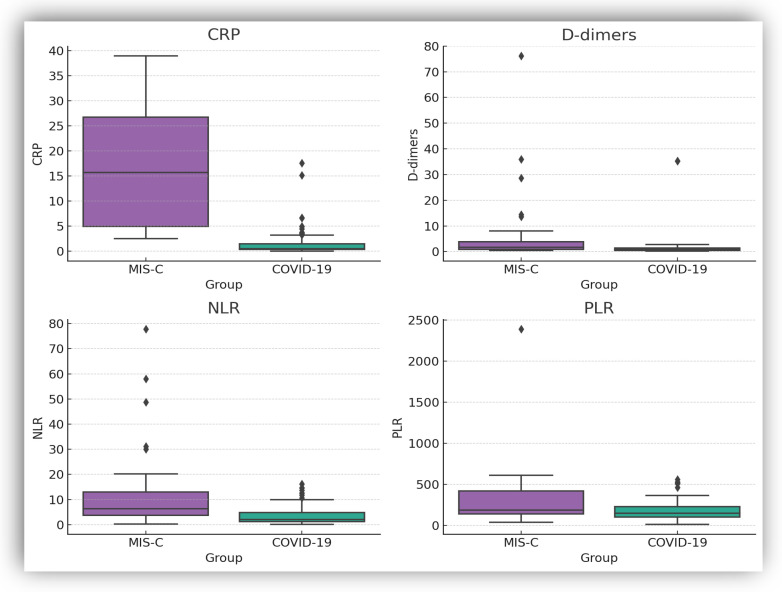
Comparison of inflammatory and hematological biomarkers between MIS-C and acute COVID-19. Box-plots display median value and interquartile ranges; whiskers extending to 1.5 × IQR, and points beyond this range are shown as outliers (dots), representing individual extreme observations. *p*-value derived from the Mann–Whitney U test.

For selected key biomarkers (CRP, D-dimer, NLR, and PLR), box-plot representations were generated to visually illustrate intergroup differences (Figure 2).

### 3.3. Correlation Patterns Among Clinical, Laboratory and Outcome Variables

To explore the relationships between biological parameters and clinical variables, Spearman correlation matrices were generated separately for the MIS-C and COVID-19 cohorts. These analyses described the overall associations pattern among inflammatory, hematological, and metabolic markers, as well as their relationship with LOS and clinical severity.

#### 3.3.1. Global Spearman Correlation Structure in MIS-C and COVID-19

**Figure 3:**
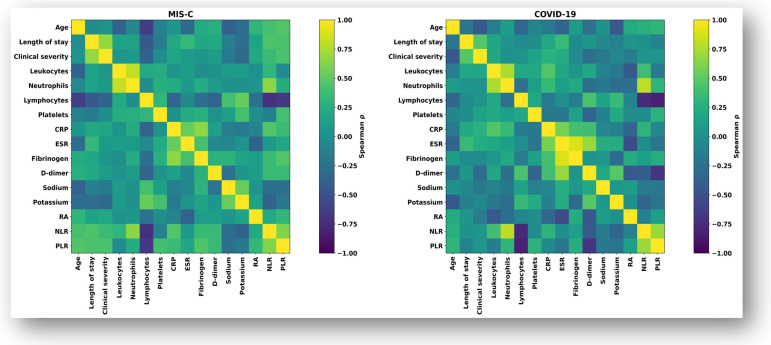
Spearman correlation heatmaps for the MIS-C and COVID-19 cohorts. Heatmaps illustrate Spearman correlation structure (ρ) across clinical and laboratory variables. Color gradients indicate the magnitude and direction of correlations. Numerical correlation coefficients are intentionally omitted to preserve figure clarity and are reported separately in Table 2.

Correlation analyses revealed distinct association profiles between clinical characteristics, laboratory parameters, and outcome variables in the MIS-C and COVID-19 cohorts. The MIS-C cohort exhibited a denser, more interconnected correlation structure involving inflammatory markers, hematological ratios, electrolyte parameters, and clinical variables, including disease severity, age, and LOS. In contrast, fewer significant correlations were observed among these variables in the COVID-19 cohort. The overall correlation structures are illustrated in Figure 3, while statistically significant associations are summarized in Table 2.

#### 3.3.2. FDR-Adjusted Spearman Correlations

To control for multiple testing, Spearman rank correlation coefficients were adjusted using the FDR procedure. FDR-adjusted correlations for the MIS-C and COVID-19 cohorts are summarized in Table 3 and Table 4, respectively.

After FDR adjustment, several strong correlations remained statistically significant in the MIS-C cohort, including associations between neutrophils and leukocytes, CRP and fibrinogen, and derived hematological indices (NLR and PLR). Inverse correlations between lymphocytes and inflammatory ratios (NLR and PLR), as well as the positive association between sodium and potassium, also remained statistically significant.

In the COVID-19 cohort, after FDR adjustment, the relationships between fibrinogen and ESR, NLR, and PLR, remained significant, as did those involving leukocytes with CRP and NLR, respectively. The association between clinical severity and LOS also remained statistically robust. In contrast, the initially observed inverse relationships between potassium and derived markers (NLR, PLR) did not meet the FDR threshold.

### 3.4. Machine-Learning-Based Predictive Modelling for MIS-C Differentiation

This analysis aimed to identify clinically relevant biomarkers with discriminatory value for differentiating MIS-C from acute COVID-19 and to evaluate the performance of the predictive models. Multivariate modelling included penalized regression and Random Forest classification, to integrate biological and clinical parameters.

#### 3.4.1. Differential Pareto Profiling of Biological Abnormalities

**Figure 4:**
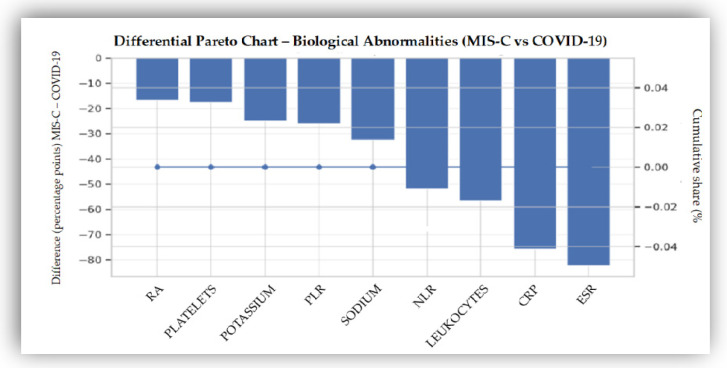
Differential Pareto chart of biological abnormalities in MIS-C versus COVID-19. Bars represent differences in the prevalence of abnormal laboratory values between cohorts (MIS-C minus COVID-19), ordered in descending magnitude. The cumulative line illustrates the aggregated contribution of individual markers and is shown for descriptive purposes only.

The distribution of biological abnormalities between the MIS-C and COVID-19 cohorts was assessed using a differential Pareto analysis. This exploratory approach provided an overview of relative differences in the prevalence of abnormal laboratory values between groups and was used solely for descriptive purposes (Figure 4). The Pareto analysis did not inform variable selection for subsequent predictive models.

Larger absolute differences in the prevalence of abnormal values were observed for inflammatory markers, including ESR and CRP, as well as for hematological parameters such as NLR and leukocyte count. In contrast, serum electrolytes (sodium, potassium) and thrombotic indicators (PLR and platelets count) showed more modest but still consistent contrasts.

#### 3.4.2. Univariate Receiver Operating Characteristic Screening of Individual Biomarkers

Univariate receiver operating characteristic (ROC) analysis was performed for each biological variable included in the study as an exploratory screening approach. For each marker, the area under the curve (AUC), the optimal threshold determined by the Youden index, and the corresponding classification parameters were calculated. It is important to note that no single biomarker demonstrated sufficient diagnostic accuracy on its own, and the univariate ROC analysis was not intended for diagnostic use.

Univariate ROC analysis yielded AUC values ranging from 0.50 to 0.75 across individual biomarkers. The highest AUC values were observed for PLR, NLR, lymphocyte count, potassium, and CRP (Table 5).

Univariate ROC analysis was used exclusively as an exploratory screening step and not for diagnostic or variable selection purposes. Variables included in multivariate predictive models were selected based on clinical relevance and data availability. Multivariate algorithms were used to determine feature selection and variable importance.

#### 3.4.3. Penalized Logistic Regression (Ridge): Full Versus Reduced Models

Two Ridge logistic regression models were developed to assess the discriminatory performance and numerical stability of the selected biomarkers: a full model including all eligible biomarkers (*n* = 13) and a reduced model based on a compact panel of five predictors (CRP, NLR, PLR, sodium, and potassium). Missing values were handled by median imputation, and model performance was evaluated using stratified 5-fold cross-validation.

The regularization parameter (λ) was selected through cross-validation. It differed markedly between models (λ ≈ 483 for the complete model and λ ≈ 3 for the reduced model), reflecting substantial multicollinearity in the extended predictor set compared to the compact panel.

**Figure 5:**
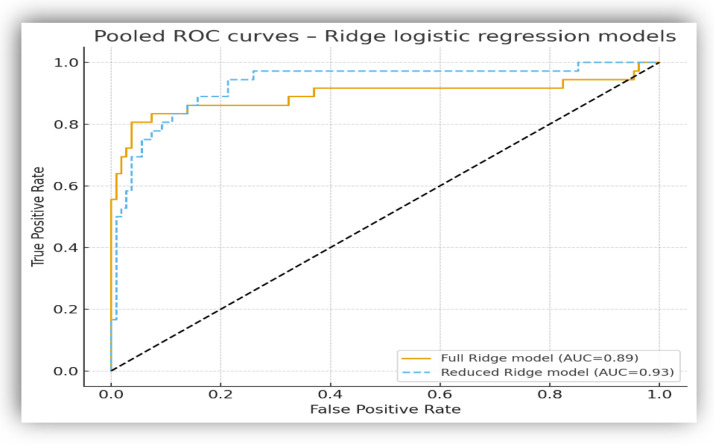
ROC curves for Ridge logistic regression models derived from stratified 5-fold cross-validation. The solid line represents the full model (13 predictors), and the dashed line represents the reduced model (5 predictors).

The complete Ridge model achieved a cross-validated AUC of 0.89 (95% CI: 0.80–0.97), while the reduced model showed a slightly higher AUC of 0.93 (95% CI: 0.87–0.97), despite the lower number of predictors (Figure 5). A standard multivariable logistic regression was also explored but yielded unstable estimates.

Multicollinearity was observed in the complete model, with high variance inflation factor (VIF) values for several predictors (Table 6), whereas the reduced model showed minimal collinearity after removal of redundant variables.

Optimal decision thresholds were derived using the Youden index on out-of-fold predictions. The full Ridge model achieved higher specificity and overall accuracy, whereas the reduced model showed higher sensitivity while maintaining robust discriminatory performance (Table 7). Both models demonstrated stable internal validation.

#### 3.4.4. Random Forest Classifier: Performance and Feature Importance

A Random Forest classifier was constructed using the five biomarkers that met the predefined eligibility criteria (CRP, potassium, sodium, NLR and PLR). Missing values were imputed using the median within a machine-learning pipeline.

**Figure 6:**
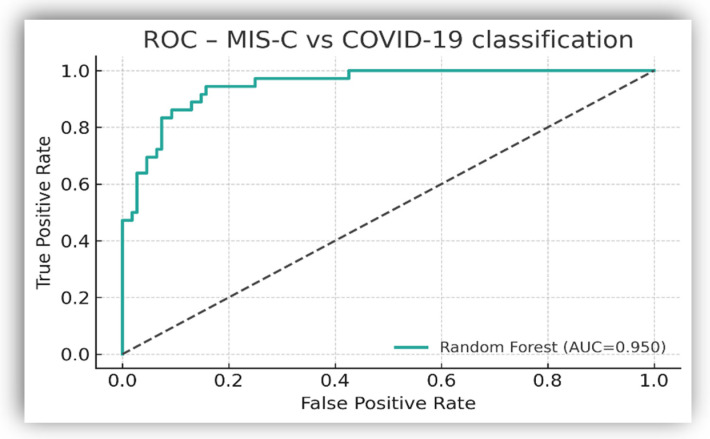
Receiver operating characteristic (ROC) curve of the Random Forest classifier distinguishing MIS-C from acute COVID-19. The cross-validated model achieved an AUC of 0.95.

Model performance was evaluated using stratified 5-fold cross-validation, yielding a AUC of 0.95 for the ROC curve (95% CI: 0.92–0.98), an overall accuracy of 0.88, a specificity of 94%, and an AUC of 0.87 for the precision-recall curve (Figure 6). Comparable performance was obtained using out-of-bag (OOB) error estimation on the full dataset (OOB accuracy: 0.88), supporting the model’s stability and arguing against overfitting.

**Figure 7:**
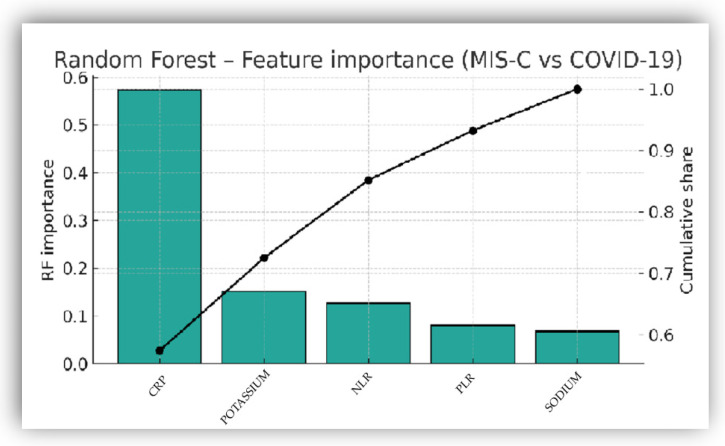
Feature importance of biomarkers included in the Random Forest classifier. The *x*-axis lists the included predictors (CRP, potassium, NLR PLR, sodium), while the *y*-axis indicates their relative importance based on the Gini index.

Feature importance was assessed using the impurity-based Gini index (Figure 7). CRP exhibited the highest relative importance (≈0.55), followed by potassium (≈0.15), NLR (≈0.16), PLR (≈0.08) and sodium (≈0.07).

Variables such as ESR and leukocyte count were not retained in the Random Forest model, as they did not meet the predefined eligibility criteria and showed redundancy with the selected predictors.

#### 3.4.5. Decision Tree Classifier: Structure, Threshold and Control Model Complexity

**Figure 8:**
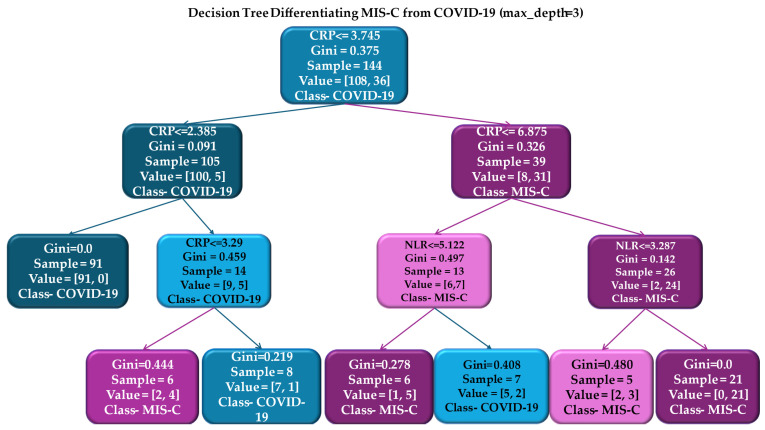
Decision tree for MIS-C versus COVID-19 classification. Each node displays the splitting variable and threshold, the Gini impurity index (a measure of node heterogeneity), the number of samples in the node, value (distribution by classes [COVID-19, MIS-C]) and class (the majority class predicted in that node). Node colors indicate the majority class predicted at each node (COVID-19 or MIS-C), with color intensity reflecting node purity (the proportion of samples belonging to the predicted class).

A decision tree classifier (CART) was developed as an interpretable control model to differentiate MIS-C from acute COVID-19 using five predefined biomarkers (CRP, NLR, PLR, sodium, and potassium). Class labels were encoded as 0 = COVID-19 and 1 = MIS-C, allowing direct visualization of the predominant diagnostic class at each terminal node (Figure 8).

CART identified CRP as the primary splitting variable. The first split occurred at CRP ≤ 3.745 mg/dL, corresponding to a branch predominantly composed of COVID-19 cases (100/108). The complementary branch (CRP > 3.745 mg/dL) included the majority of MIS-C cases, with a second CRP threshold (≈6.875 mg/dL) further stratifying this group. Within the intermediate CRP range (≈3.7–6.9 mg/dL), NLR was selected as the subsequent splitting at NLR ≤ 5.12 separating branches with differing class composition. On the higher CRP branch, a second NLR threshold (≈3.28) resulted in a terminal node with complete class homogeneity for MIS-C (Gini = 0.0).

**Figure 9:**
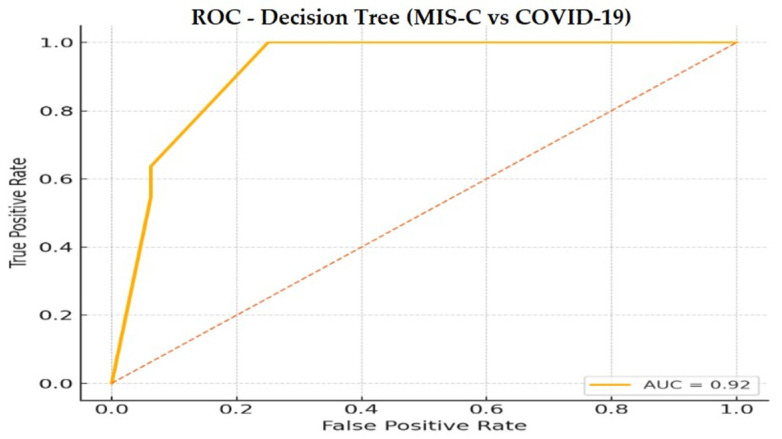
Receiver operating characteristic (ROC) curve for the Decision Tree classifier distinguishing MIS-C from acute COVID-19. Performance was evaluated using stratified 5-fold cross-validation (AUC = 0.92).

Model performance assessed by stratified 5-fold cross-validation yielded an AUC of 0.92 (95% CI: 0.86–0.96), a sensitivity of 72.2%, a specificity of 89.0%, and an overall accuracy of 85% (Figure 9).

#### 3.4.6. Exploratory Analysis of Threshold Stability Across Single-Split Decision Stumps

**Figure 10:**
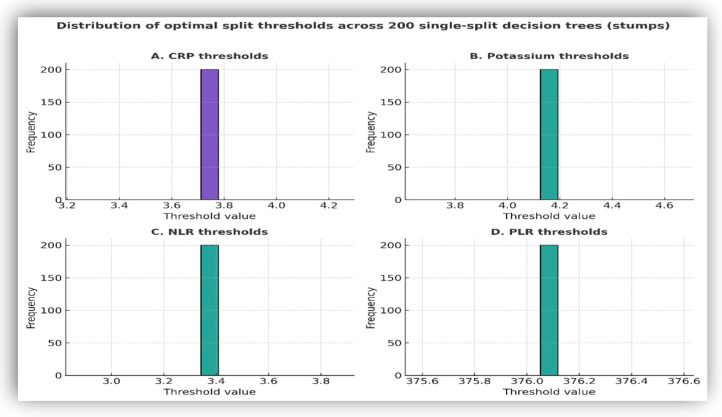
Distribution of optimal split thresholds across 200 single-split decision stumps. Each panel shows the frequency distribution of the threshold values selected for CRP, potassium, NLR, and PLR across bootstrap resampling, illustrating the consistency of cut-off values.

To assess the stability of the decision thresholds identified in tree-based models, an exploratory analysis using single-split decision stumps was performed via bootstrap resampling. This approach evaluated whether consistent cut-off values emerged when the dataset was repeatedly partitioned (Figure 10).

Across resampling iterations, CRP and NLR consistently yielded narrowly distributed thresholds, centered at approximately 3.7 mg/dL for CRP and 3.3 for NLR. Potassium and PLR showed broader and less consistent threshold distributions, while sodium and D-dimer did not generate reproducible cut-off values. ESR did not yield stable thresholds across resampling iterations and was not retained in multivariable models.

Results from descriptive, univariate, and multivariate analyses were summarized in Table 8, which provides a comparative overview of biomarker involvement across the different analytical approaches. Table 8 is intended as a descriptive synthesis and does not represent a ranking or weighting of biomarkers.

## 4. Discussion

Our findings indicate that MIS-C and pediatric COVID-19 represent distinct clinical entities with different pathophysiological trajectories, inflammatory profiles, and biological signatures, despite their shared association with SARS-CoV-2. These differences have relevant implications for differential diagnosis and clinical management in pediatric practice.

Integrated analysis of inflammatory, hematological, and electrolyte biomarkers, together with key clinical outcomes, revealed clearly differentiated biological patterns between MIS-C and pediatric COVID-19. These findings are consistent with previous reports and further support the concept of MIS-C as a distinct hyperinflammatory condition with a specific biological profile, rather than a more severe form of acute pediatric COVID-19 [39,40,41,42,43].

A multimethod analytical strategy was employed to address the complex and nonlinear biological processes underlying MIS-C. The combined use of regression-based, ensemble, and tree-based models allowed complementary evaluation of discriminatory performance, robustness, and clinical interpretability, highlighting convergent patterns across methods.

Multiple internal validation strategies, including cross-validation, out-of-bag estimation, and threshold stability analyses, supported model robustness. The convergence of results across these independent approaches reduces the likelihood of model-specific artefacts and strengthens confidence in the reported findings.

### 4.1. Biological Profile and Comparative Analysis of MIS-C Versus COVID-19

The comparative analysis confirmed a markedly more severe clinical course in MIS-C compared to pediatric COVID-19, reflected by higher disease severity and substantially longer LOS. Similar patterns have been consistently reported in pediatric cohorts, in which prolonged LOS in MIS-C cases has been attributed to multisystem involvement and intense systemic inflammation [44].

From a biological perspective, MIS-C was characterized by a hyperinflammatory phenotype, in line with recent meta-analytical data describing amplified inflammatory and hematological responses compared to pediatric COVID-19 [45,46].

Previous studies have further highlighted the value of hematological indices derived as differential markers, reporting higher NLR and related indices in MIS-C than in COVID-19 [42]. In pediatric COVID-19 cohorts, NLR has been consistently associated with disease severity, whereas PLR has shown more heterogeneous or weaker associations, suggesting that these ratios capture related but non-identical inflammatory processes [35,36]. In MIS-C, this inflammatory signal appears amplified and more tightly integrated, extending beyond the ranges typically reported in pediatric cohorts with symptomatic COVID-19, as reflected in our cohort by lymphopenia and changes in the platelet-to-lymphocyte ratio, findings consistently reported in pediatric inflammatory cohorts [36,47,48].

These hematological features reflect a profoundly dysregulated immune profile in MIS-C, characterized by amplified neutrophilic activation and disturbances of the adaptive immune response [49]. Recent immunological studies have described CD8+ T-cell hyperactivation and expansion of the vascular-patrolling CX3CR1+ CD8+ T-cell subset as hallmark features of MIS-C [50,51,52,53]. In contrast, acute pediatric COVID-19 is associated with a more restricted inflammatory response, dominated by moderate hematological changes and lacking consistent multisystem involvement [51,52].

Beyond inflammatory markers, differences between cohorts also extended to electrolyte balance, with sodium and potassium showing significant variations in MIS-C. Similar disturbances, including hyponatremia and hypokalemia, have been reported in association with severe inflammatory states and hyperinflammatory syndromes [31,37,54]. In MIS-C, these electrolyte disturbances may be driven by systemic inflammation, particularly through IL-6-mediated effects on ADH secretion and fluid balance, mechanisms that are well documented in inflammatory states [55,56,57,58].

However, clinical management factors may also influence electrolyte values in hospitalized children. Fluid rebalancing, intravenous therapies, and the timing of laboratory sampling could partially contribute to the observed variations.

### 4.2. Correlation Patterns Between Biomarkers and Clinical Parameters in MIS-C and COVID-19 Cohorts

#### 4.2.1. Correlational Architecture in the Studied Cohorts

In the MIS-C cohort, the correlational architecture was denser and more coherent than in the COVID-19 cohort, reflecting the simultaneous activation of multiple inflammatory pathways.

In MIS-C, prolonged LOS was associated with increased disease severity and inflammatory hematological disturbances, including elevated NLR and PLR, higher ESR, and lymphopenia. These results indicate that hematological abnormalities are closely linked to clinical evolution rather than representing isolated laboratory findings. While previous studies have primarily emphasized cardiac involvement and intensive care requirements in MIS-C [59,60,61], the present results highlight the relevance of routinely available inflammatory indices in capturing the systemic nature of the disease. Similar associations between elevated NLR and disease severity, as well as correlations with LOS, have been reported in pediatric COVID-19 cohorts. In contrast, PLR has shown more heterogeneous associations with clinical severity, suggesting that these ratios reflect related but not identical inflammatory processes across pediatric and adult COVID-19 populations [36,48,62].

The observed associations between CRP, NLR, and PLR delineate an integrated inflammatory core characteristic of an active systemic process, consistent with established MIS-C pathophysiology marked by neutrophilia, lymphopenia, and adaptive immune dysregulation [42,45,63,64,65,66]. This coordinated pattern of neutrophilic and platelet activation has been proposed as a sensitive indicator of MIS-C in both clinical and translational studies [49,62].

Although descriptive analysis revealed moderate differences in electrolytes (sodium and potassium), correlational models showed positive mutual associations and inverse relationships with NLR and PLR, a pattern also reported by Dalal et al. (2023) [67]. These findings suggest that electrolyte changes are integrated into the global inflammatory network of MIS-C, reflecting the interaction between volume status and inflammatory intensity [48,54].

In contrast, pediatric COVID-19 exhibited a simpler correlational architecture, limited primarily to traditional inflammatory relationships involving CRP, leukocytes, and NLR. Disease severity showed only modest associations with age, as previously reported in pediatric cohorts [68], while LOS displayed weak relationships with most biological parameters. This narrower, less integrated inflammatory network highlights fundamental biological differences between MIS-C and pediatric COVID-19, with MIS-C exhibiting a more complex, interdependent inflammatory profile [10,69,70].

#### 4.2.2. FDR Analysis and Robust Association Patterns

After FDR adjustment, only associations involving key inflammatory markers and inverse relationships with lymphocyte counts remained statistically significant in the MIS-C cohort. This finding supports the presence of a compact and biologically coherent inflammatory core, rather than spurious correlations arising from multiple testing. These robust associations are consistent with established immunological features of MIS-C, including neutrophilia, hyperfibrinogenemia, elevated composite inflammatory ratios, and marked lymphopenia. Collectively, these alterations reflect disproportionate innate immune activation accompanied by suppression of adaptive immune responses [50,62,71,72].

In contrast, FDR adjustment in the pediatric COVID-19 cohort retained only associations typical of acute inflammation, highlighted by relationships between CRP, leukocyte counts, and NLR. Electrolyte- and thrombosis-related markers did not demonstrate robust associations after correction, suggesting that inflammatory involvement in pediatric COVID-19 is more limited and focal, without the systemic network activation characteristic of MIS-C [73,74,75].

Together, the robust correlation patterns identified by Spearman and FDR-adjusted analyses, combined with the associations between inflammatory markers and LOS, support the relevance of CRP, NLR, PLR, and selected electrolyte parameters in delineating disease severity and biological differences between MIS-C and COVID-19 [36].

### 4.3. Convergence of Predictive Methods and Delineation of the MIS-C Biological Signature

Integration of the predictive methods applied in this study revealed a stable biological signature, with the same biomarkers consistently emerging as the most discriminatory for MIS-C compared with pediatric COVID-19. Across analytical frameworks, CRP and NLR showed the strongest and most reproducible signals, while PLR and electrolyte parameters provided additional but more modest contributions. The recurrence of these markers across models suggests genuine pathophysiological differences rather than methodological artefacts and supports their potential role in early clinical risk stratification, pending external validation.

This convergent discriminatory pattern is clinically relevant, as early MIS-C and acute COVID-19 often overlap at onset. The persistence of the same biomarkers across multiple analytical strategies indicates that the models capture reproducible physiological distinctions rather than merely reflecting predefined diagnostic criteria.

Previous multicenter studies have consistently reported increased CRP and D-dimer levels in MIS-C, together with alterations in derived indices such as NLR and PLR, supporting their value as markers of severity in pediatric inflammatory disorders [42,65]. Our findings are concordant with this pattern. However, D-dimer contributed less to multivariable models, which may reflect early anticoagulant administration in the MIS-C cohort. This issue was also acknowledged in recent literature and potentially attenuating their discriminatory performance in adjusted analyses [44,76].

The Pareto analysis provided complementary support for this biological signature by highlighting marked differences in prevalence across key inflammatory markers, particularly ESR, CRP, and NLR. Unlike multivariate models, this approach emphasizes the frequency of abnormalities rather than their absolute values, allowing identification of markers with pronounced differential prevalence between cohorts [44,63].

Univariate ROC analyses further supported this pattern, with PLR and NLR showing the highest individual discriminatory performance, reflected by AUC values in the moderate-to-good range (approximately 0.75 and 0.71, respectively). At the same time, other biomarkers demonstrated more limited standalone accuracy, a pattern consistently reported in predictive analyses of inflammatory ratios in COVID-19 and MIS-C cohorts [35,77,78,79]. This observation is consistent with the complex and highly collinear pathophysiology of MIS-C, in which markers with modest individual performance may become informative when integrated into multivariate models [50,71].

Recent immunological studies provide mechanistic context for these findings, describing marked activation of CD8^+^ CX3CR1^+^ T cells in MIS-C, a subset involved in vascular surveillance and endothelial interaction [39,50,80,81]. Activation of the CX3CR1–CX3CL1 axis has been linked to vascular dysfunction and thromboinflammatory manifestations, supporting the concept of an exaggerated immune response distinct from that observed in pediatric acute COVID-19 [31,82,83]. In addition, increased expression of T-cell activation and exhaustion markers, such as PD-1 and CD39, together with persistent antigenic stimulation, supports the hypothesis of a prolonged and amplified immune response characteristic of MIS-C [39,80,84].

Overall, the convergence of biomarkers identified across complementary analytical approaches highlights the presence of a stable and biologically coherent MIS-C signature. This integrative pattern adds to the existing literature by linking inflammatory, hematological, and electrolyte markers within a unified, data-driven framework.

### 4.4. Multivariate Models and the Contribution of Biomarkers in MIS-C Prediction

Multivariate modelling confirmed that a restricted panel of routinely available biomarkers is sufficient to discriminate MIS-C from pediatric COVID-19. Across predictive approaches, both Random Forest and reduced Ridge models consistently converged on the same five predictors—CRP, NLR, PLR, sodium, and potassium—highlighting a compact and reproducible discriminatory signature.

Penalized logistic regression (Ridge) is increasingly used in pediatric clinical research to stabilize inference in the presence of moderate sample sizes and collinear predictors. This approach is widely recommended in clinical prediction modeling and has demonstrated applicability in pediatric research for stabilizing inference and integrating correlated clinical and biological markers into practical clinical decision-support frameworks [35,85,86]. Notably, in our dataset, the reduced five-predictor model outperformed the complete model (AUC 0.93 vs. 0.89), supporting the concept that compact biomarker panels can retain strong discriminatory capacity when redundant variables are removed.

Although D-dimer showed marked elevation and prevalence differences, it did not add incremental value in multivariable models once CRP and NLR were included [36,77]. This behavior likely reflects redundancy with dominant inflammatory pathways rather than a lack of biological significance, a phenomenon previously reported in pediatric inflammatory cohorts [31,59,83,87,88].

It is important to emphasize that Ridge regression cannot demonstrate predictor independence. For this reason, classical logistic regression was explored but not retained, as coefficient instability due to increased collinearity—well recognized in inflammatory biomarker panels—limited interpretability. VIF analysis of the extended model confirmed this issue, with sodium, fibrinogen, leukocytes, and neutrophils exhibiting very high VIF values. Since collinearity primarily affects larger models, and the reduced five-predictor panel was selected based on clinical relevance and a robust discriminatory signal, further VIF evaluation was not required.

Tree-based models provided a complementary perspective, independent of assumptions about linear parameters. Random Forest consistently identified CRP as the leading predictor, followed by potassium, NLR, and PLR [40,50,67,71,78,79,83,89]. ESR and leukocyte count were not retained due to limited incremental discriminatory value, redundancy with CRP/NLR, and numerical instability [90,91].

The decision tree generated simple, clinically interpretable rules, predominantly based on CRP and NLR. The CRP thresholds identified by the model (approximately 3.7 mg/dL and 6.9 mg/dL) fall within biologically meaningful ranges corresponding to escalating inflammatory severity in MIS-C [54,92,93]. Although these thresholds are not intended to reproduce diagnostic criteria, their proximity to values commonly used in MIS-C definitions (e.g., CRP ≥ 3 mg/dL in WHO/ECDC guidelines) suggests that the algorithm captures genuine pathophysiological patterns rather than arbitrary statistical divisions [28].

Threshold stability analyses further supported these findings, with CRP and NLR showing the most reproducible cut-offs, and potassium and PLR contributing secondary but consistent discriminatory signals. This pattern is consistent with the complementary roles of univariate and multivariate approaches whereby marginal discriminatory signals may not necessarily generate splits in fully multivariate models. Potassium values largely remained within pathophysiological ranges. However, their systematic shift toward lower values in MIS-C reflects subtle metabolic involvement, likely linked to inflammation-related fluid regulation and activation of the IL-6–ADH axis, as described in inflammatory and post-infectious pediatric conditions [61,67,94]. Thus, potassium does not function as an isolated severity marker, but as a complementary differentiating variable, leveraged by algorithmic models when inflammatory markers approach decisional thresholds.

Among all hematologic variables evaluated, NLR and PLR demonstrated the highest degree of methodological robustness [35,74,75]. Their relevance was independently confirmed across three analytical dimensions: statistical association after FDR adjustment, model-based importance in Ridge regression and Random Forest under internal cross-validation, and threshold stability in bootstrap decision stumps. Few biomarkers evaluated in MIS-C research show comparable reproducibility. Their behavior reflects the characteristic pathophysiological triad of neutrophilia, lymphopenia, and platelet activation, supporting their role as reliable markers for early differentiation between MIS-C and pediatric COVID-19 [63,64,65,66,73,95].

The stability of the thresholds obtained through bootstrap resampling provides an additional argument for their potential use. The recurrence of cut-off values for CRP (~3.7 mg/dL), potassium (~4.2 mmol/L), NLR (~3.3), and PLR (~376) indicates not only methodological robustness but also practical applicability in rapid triage contexts.

However, these findings should be interpreted in the context of a single-center, retrospective study design. While the identified biomarker panel and threshold-based models demonstrated strong internal consistency and biological plausibility, they should be regarded as exploratory and hypothesis-generating. External validation in independent, multicenter cohorts and prospective studies is required before translation into routine clinical practice.

Overall, the convergence of penalized regression, ensemble algorithms, decision trees, and prevalence-based analyses demonstrates that MIS-C is not merely a severe manifestation of pediatric COVID-19, but a distinct post-infectious immunological entity. This condition is characterized by profound systemic inflammation, marked hematological imbalances, and metabolically relevant abnormalities. The development of simplified, reproducible biomarker-based algorithms represents a promising direction, although further validation is required before clinical implementation.

Our study has several limitations that should be considered when interpreting the results. First, the retrospective observational design limits the ability to establish causal relationships and is inherently exposed to incomplete or unevenly documented data. The relatively small sample size, particularly within severity subgroups, and the single-center design represent additional limitations.

In addition, the wide pediatric age range (0–18 years) introduces clinical heterogeneity, as disease presentation and severity may vary across developmental stages. However, restricting the age interval would have substantially reduced the number of available MIS-C cases and limited statistical power. The chosen approach reflects real-world pediatric clinical practice.

Although the set of analyzed variables was limited, it was not possible to control all potential factors influencing biomarker levels, such as hydration status, fluid administration, or timing of anti-inflammatory treatment initiation. Another significant limitation is that biomarker assessment was performed only during hospitalization, without post-discharge follow-up, precluding evaluation of long-term inflammatory, hematological, or electrolyte dynamics.

A relevant methodological limitation is the inability to stratify COVID-19 cases by circulating viral variants (e.g., Alpha, Delta, Omicron), which limited the assessment of potential variant-specific pathophysiological differences, including electrolyte disturbances and variations in hematological indices.

The present findings should also be interpreted in the context of external validity. As this was a single-center retrospective study conducted in a regional pediatric population, generalizability to other settings may be limited. Nevertheless, the consistency of the results across multiple analytical approaches supports their biological plausibility, and external validation in independent multicenter cohorts is warranted.

These limitations, inherent to retrospective analyses, do not invalidate the findings but underscore the need for prospective, multicenter studies with longitudinal follow-up to validate and extend the conclusions presented.

### 4.5. Future Perspectives

The results of this study open several relevant research directions, both clinically and methodologically. The convergence of biomarkers identified by different predictive approaches—penalized regression, Random Forest, decision trees, and threshold-based models—suggests a robust biological signature that warrants validation in larger, multicenter cohorts and across diverse clinical settings. Such validation would allow the establishment of generalizable thresholds and their translation into standardized diagnostic tools.

The stability of derived inflammatory markers (NLR, PLR), together with the complementary contribution of electrolytes, highlights the potential development of a simple clinical mini-score or rapid triage algorithm applicable in emergency settings. Prospective evaluation of such tools could shorten diagnostic delays and improve early patient management.

From a pathophysiological perspective, the dense correlational phenotype observed in MIS-C suggests a highly interconnected inflammatory network. Future studies should explore the integration of routinely available biomarkers with advanced immunological parameters, such as cytokine profiles, lymphocyte subsets, and endothelial activation markers, to better define disease-specific immune signatures and clarify the mechanisms that distinguish MIS-C from acute COVID-19.

Longitudinal investigations are also needed to assess the persistence of hematological and electrolyte abnormalities and to determine their potential role in predicting long-term sequelae. Such data would directly inform post-discharge monitoring strategies and the development of standardized follow-up protocols.

From a technological standpoint, the strong performance of machine-learning models, even with relatively small datasets, supports the development of computerized alert systems integrated into clinical laboratory workflows. These systems could facilitate early identification of biological patterns suggestive of MIS-C and support real-time clinical decision-making.

Finally, as early therapeutic interventions (immunoglobulins, corticosteroids, anticoagulants) may influence biomarker levels, future studies should distinguish between markers reflecting disease presentation and those modified by treatment. This distinction is essential for the development of diagnostic and prognostic algorithms that remain robust across different therapeutic strategies.

Overall, these perspectives emphasize the need to integrate clinical, immunological, and computational approaches to advance the understanding of MIS-C and to develop predictive tools with tangible impact on pediatric care.

## 5. Conclusions

The present study demonstrates that MIS-C and pediatric COVID-19 do not represent a continuum of disease severity, but rather distinct biological entities governed by fundamentally different immunological and inflammatory mechanisms. Through integrated descriptive, correlational, and predictive analyses, we identified a reproducible MIS-C biological signature centered on a compact panel of routinely available biomarkers—CRP, NLR, PLR, sodium, and potassium—that consistently demonstrated strong discriminative performance across all evaluated models.

The stability of these biomarkers and the recurrence of clinically meaningful thresholds suggest that they capture core elements of MIS-C pathophysiology, distinct from the more limited inflammatory response observed in pediatric COVID-19. In this context, the use of a narrow and easily accessible laboratory panel may facilitate rapid triage, early risk stratification, and timely clinical decision-making in pediatric settings.

By combining methodological rigor with clinical applicability, this study provides a foundation for developing standardized differential diagnostic tools. It supports future research directions focused on multicenter validation, expansion of immunological profiling, and the construction of unified post-COVID pediatric monitoring algorithms. Overall, early identification of MIS-C may be optimized by using a compact biomarker core with high, reproducible predictive consistency.

## Figures and Tables

**Table 1:** Comparative analysis of routine laboratory biomarkers in MIS-C and COVID-19.

Biomarker	MIS-C (*n* = 36) Median (IQR)	COVID-19 (*n* = 108) Median (IQR)	*p*-Value
CRP [mg/dL]	15.69 [4.92–26.72]	0.5 [0.35–1.51]	<0.001
ESR [mm/h]	60.5 [29.5–94.0]	10.0 [8.0–20.0]	<0.001
D-dimer [ng/mL]	1.72 [0.85–3.87]	0.93 [0.42–1.46]	0.0080
Fibrinogen [mg/dL]	443.0 [273.70–622.00]	262.5 [196.5–333.5]	<0.001
Leukocytes [10^3^/μL]	15.07 [12.55–22.07]	9.15 [6.47–12.66]	<0.001
Neutrophils [10^3^/μL]	11.37 [7.60–14.12]	4.92 [3.31–8.37]	<0.001
Lymphocytes [10^3^/μL]	1.64 [1.03–2.54]	2.22 [1.37–3.04]	0.0359
Platelets [10^3^/μL]	273.8 [209.62–583.85]	321.75 [277.65–409.85]	0.3204
NLR [ratio]	6.33 [3.69–13.00]	2.02 [1.20–4.75]	<0.001
PLR [ratio]	185.39 [138.32–420.17]	150.29 [101.10–228.87]	0.0242
Sodium [mmol/L]	135 [133–137]	136 [135–139]	0.0103
Potassium [mmol/L]	4.01 [3.66–4.57]	4.45 [4.12–4.90]	0.0127
AR [mmol/L]	22 [18.75–25]	22 [20–25]	0.3398

Footnote: Data are presented as median (IQR); *p*-value were calculated using the Mann–Whitney U test. A two-sided *p*-value < 0.05 was considered statistically significant.

**Table 2:** Statistically significant Spearman correlations between clinical, laboratory and outcome variables in the MIS-C and COVID-19 cohorts.

Cohort	Variable 1	Variable 2	Spearman ρ
MIS-C	LOS	Clinical severity	0.69
MIS-C	LOS	NLR	0.44
MIS-C	LOS	PLR	0.43
MIS-C	LOS	ESR	0.33
MIS-C	LOS	Lymphocytes	−0.44
MIS-C	NLR	PLR	0.62
MIS-C	CRP	NLR	0.46
MIS-C	Sodium	Potassium	0.54
COVID-19	Clinical severity	Age	0.30
COVID-19	LOS	ESR	0.35
COVID-19	Leukocytes	CRP	0.46
COVID-19	Leukocytes	NLR	0.38
COVID-19	CRP	NLR	0.30
COVID-19	NLR	PLR	0.70
COVID-19	Potassium	NLR	−0.35
COVID-19	Potassium	PLR	−0.22

Footnote: Only statistically significant correlations are reported. Spearman correlation analysis was performed separately for the MIS-C and COVID-19 cohorts. Abbreviations: LOS, length of hospitalization (days); clinical severity, ordinal disease severity category.

**Table 3:** FDR-adjusted Spearman correlations in the MIS-C cohort.

Variable 1	Variable 2	Spearman ρ	pFDR
Neutrophils	Leukocytes	0.81	1.3 × 10^−7^
Lymphocytes	NLR	−0.70	6.7 × 10^−5^
LOS	Clinical severity	0.69	9.6 × 10^−5^
Lymphocytes	PLR	−0.67	1.75 × 10^−4^
CRP	Fibrinogen	0.66	7.9 × 10^−3^
NLR	PLR	0.62	6.1 × 10^−4^
Sodium	Potassium	0.54	7.0 × 10^−3^
CRP	ESR	0.53	7.0 × 10^−3^

**Table 4:** FDR-adjusted Spearman correlations in the COVID-19 cohort.

Variable 1	Variable 2	Spearman ρ	pFDR
ESR	Fibrinogen	0.78	2.1 × 10^−6^
NLR	PLR	0.70	1.1 × 10^−5^
Leukocytes	CRP	0.46	2.0 × 10^−3^
Leukocytes	NLR	0.38	7.0 × 10^−3^
LOS	Clinical form	0.31	3.1 × 10^−3^
CRP	NLR	0.30	4.6 × 10^−2^

Abbreviations: pFDR, false discovery rate-adjusted *p*-value (Benjamini–Hochberg).

**Table 5:** Performance of individual biomarkers in univariate ROC analysis (MIS-C versus COVID-19).

Biomarker	AUC	Optimal Threshold (Cut-Off)	Rule	Sensitivity	Specificity	Accuracy
PLR	0.75	331.03	≥cut-off	55%	94%	72%
NLR	0.71	8.57	≥cut-off	55%	88%	69%
Lymphocytes	0.71	1.32	≤cut-off	60%	94%	75%
Potassium	0.68	3.64	≤cut-off	40%	94%	64%
AR	0.62	25.0	≥cut-off	50%	81%	64%
CRP	0.61	15.67	≥cut-off	65%	62%	64%
Sodium	0.61	136.0	≤cut-off	85%	44%	67%
D-dimer	0.57	8.07	≥cut-off	25%	94%	56%
Hemoglobin	0.56	10.23	≤cut-off	45%	81%	61%
ESR	0.55	67.0	≥cut-off	55%	62%	58%
Leukocytes	0.53	28.45	≥cut-off	20%	100%	56%
Fibrinogen	0.53	294.0	≥cut-off	84%	45%	70%
Platelet count	0.50	209.7	≤cut-off	40%	88%	61%

Footnote: Optimal threshold, value determined by the Youden index; rule, direction of classification (≥ or ≤cut-off).

**Table 6:** VIF value (full model).

Predictor	VIF
Sodium	65.3
AR	49.1
Leukocytes	28.2
Neutrophils	18.9
Fibrinogen	19.9
Platelet count	12.6
PLR	9.9
NLR	8.6
Lymphocytes	6.0
ESR	5.6
CRP	3.9
Potassium	1.4
D-dimer	1.3

**Table 7:** Performance metrics based on the Youden index for Ridge logistic regression models.

Metric	Value Full Ridge	Value Reduced Ridge Model
Optimal threshold (Youden)	0.257	0.158
Sensitivity	80.6%	88.9%
Specificity	96.3%	84.3%
Accuracy	92.4%	85.4%

**Table 8:** Summary of biomarker relevance across all analytical approaches.

Biomarker	Pareto	Random Forest	Ridge (Reduced Model)	Decision Tree (Split Variable)	Stumps Analysis
CRP [mg/dL]	+43 pp	Highest	Included	Yes (3.745/6.875)	~3.7
NLR [ratio]	+40 pp	High	Included	Yes (5.12/3.28)	~3.3
ESR [mm/h]	+45 pp	Not selected	Not included	-	-
D-dimer [mg/dL]	moderate	Not selected	Not included	-	–
Potassium [mmol/L]	low–moderate	Moderate	Included	No split	~4.2
Sodium [mmol/L]	low	Low	Included	No split	-
PLR [ratio]	low–moderate	Low	Included	No split	~376

Footnote: pp = percentage points; Threshold values reported in the stumps analysis represent the most frequently observed split values across bootstrap resampling.

## Data Availability

Personal medical data are publicly unavailable due to privacy or ethical restrictions, being obtained from the medical record of the patient admitted into “St. John” Emergency Clinical Hospital for Children in Galati, Romania. De-identified data supporting the findings are available from the first author upon reasonable request and with institutional approval.

## Supplementary Materials

The following supporting information can be downloaded at: https://www.mdpi.com/article/10.3390/biomedicines14020258/s1, Supplementary Methods containing additional methodological details regarding the statistical and machine-learning analyses.

## Abbreviations

The following abbreviations are in this manuscript:

AR	Alkaline reserve (synonym: ECO_2_/bicarbonate)
AUC	Area Under the Curve
ADH	Antidiuretic hormone
CART	Classification and regression Trees
CDC	Centers for Disease Control and Prevention
COVID-19	Coronavirus Disease 2019
CRP	C-reactive protein
CTSE	Council of State and Territorial Epidemiologists
ECDC	European Centre for Disease Prevention and Control
ESR	Erythrocyte sedimentation rate
FDR	False discovery rate (Benjamini–Hochberg)
IgG	Immunoglobulin G
IgM	Immunoglobulin M
IL-6	Interleukin 6
IL-1β	Interleukin 1 beta
IQR	Interquartile range
LOS	Length of hospitalization
MIS-C	Multisystem inflammatory syndrome in children, associated with COVID-19
NLR	Neutrophil-to-lymphocyte ratio
PLR	Platelet-to-lymphocyte ratio
pFDR	False discovery rate-adjusted p-value (Benjamini–Hochberg)
ROC	Receiver Operating Characteristic
RNA	Ribonucleic acid
qRT-PCR	Quantitative reverse transcription polymerase chain reaction
SARS-CoV-2	Severe Acute Respiratory Syndrome-Related Coronavirus 2
VIF	Variance Inflation Factor
WHO	World Health Organization
