# Supplementary material for: Differentiating Multisystem Inflammatory Syndrome in Children (MIS-C) from Acute COVID-19 Using Biomarkers: Toward a Practical Clinical Scoring Model
Source: Biomedicines. 2026 Jan 23;14(2):258. doi: 10.3390/biomedicines14020258 (PMC12937947; doi:10.3390/biomedicines14020258)
Supplement: Supplementary file 1 [file biomedicines-14-00258-s001.zip › biomedicines-4063935-supplementary.pdf]

SUPPLEMENTARY METHODS

Article

Differentiating Multisystem Inflammatory Syndrome in Children (MIS-C) from Acute COVID-19 Using Biomarkers: Toward a Practical Clinical Scoring Model

Carmen Loredana Petrea (Cliveți) [1,2,+], Diana-Andreea Ciortea [1,3,*], Gabriela Gurău [1,2,*], Mădălina Nicoleta Matei [1,2,+], Alina Plesea Condratovici [1], Andreea Eliza Zaharia [1,2], Codrina Barbu (Ivașcu) [1,2], Gabriela Isabela Verga (Răuță) [1,2] and Sorin Ion Berbece [1]

S1. Data preprocessing and handling of missing values

Continuous laboratory variables were assessed for completeness prior to analysis. Missing values were infrequent and were handled using **median imputation**, applied within the machine-learning pipeline to avoid data leakage between training and validation sets. Imputation was performed separately within each resampling iteration.

Derived hematological ratios were calculated as follows:

- Neutrophil-to-lymphocyte ratio (NLR) = absolute neutrophil count / absolute lymphocyte count
- Platelet-to-lymphocyte ratio (PLR) = platelet count / absolute lymphocyte count

No additional data transformations or normalization procedures were applied, as tree-based models are invariant to monotonic scaling.

S2. Biomarker selection for multivariate and machine-learning analyses

Prior to multivariate and machine-learning modelling, candidate biomarkers were selected using predefined eligibility criteria to ensure clinical relevance, interpretability, and numerical stability. Biomarker selection was not data-driven alone but guided by both biological plausibility and methodological considerations.

Specifically, candidate variables were evaluated according to four criteria: (1) **clinical and biological plausibility**, based on known inflammatory, immune, or cardiovascular involvement in MIS-C and acute COVID-19; (2) **sufficient data completeness**, allowing reliable statistical estimation; (3) **evidence of discriminatory potential** in preliminary descriptive analyses, including differential Pareto profiling and univariate ROC screening; and

(4) **numerical stability**, avoiding extreme or sparsely distributed values that could bias multivariate or machine-learning algorithms.

Only biomarkers meeting all four criteria were retained for inclusion in multivariate and machine-learning models. This strategy ensured that subsequent analyses were performed on variables with both biological interpretability and robust discriminatory potential, while minimizing instability related to collinearity or data sparsity.

S3. Differential Pareto analysis

A differential Pareto analysis was performed to visually compare the prevalence of abnormal laboratory values between the MIS-C and COVID-19 cohorts. Laboratory values were dichotomized as normal or abnormal according to age-specific pediatric reference ranges.

This analysis was used exclusively for descriptive purposes, to illustrate relative differences in the frequency of biological abnormalities between groups. It was not used for feature selection, threshold derivation, or model development.

S4. Univariate ROC screening

Univariate receiver operating characteristic (ROC) analyses were conducted as an exploratory screening approach to evaluate the individual discriminatory performance of each biological variable. For each marker, the area under the ROC curve (AUC) was calculated, along with the optimal cut-off value determined using the Youden index.

Sensitivity, specificity, and related classification parameters were reported descriptively. As stated in the main manuscript, no single biomarker demonstrated sufficient standalone diagnostic accuracy, and univariate ROC analysis was not intended for diagnostic use.

S5. Penalized logistic regression (Ridge)

Penalized logistic regression using L2 regularization (Ridge regression) was applied to reduce coefficient instability in the presence of collinearity among inflammatory biomarkers. Model tuning was performed using internal cross-validation to select the optimal regularization parameter.

Both full and reduced models were evaluated. The reduced model retained only predictors showing consistent discriminatory signals across analytical approaches. Model performance was assessed using ROC AUC and internal cross-validation.

S6. Random Forest classification

Random Forest models were trained using an ensemble of decision trees constructed on bootstrap-resampled datasets. Variable importance was assessed using **mean decrease in Gini impurity**. Model performance was evaluated using **stratified 5-fold cross-validation** and **out-of-bag (OOB) error estimation**.

Random Forest was selected due to its ability to capture nonlinear relationships and interactions without assumptions regarding variable distribution.

S7. Decision tree modeling and threshold stability

Single decision tree models were generated to provide **clinically interpretable classification rules**. To assess the robustness of decision thresholds, **bootstrap-based single-split decision stumps** were constructed across repeated resampling iterations.

Threshold stability was evaluated by examining the distribution of cut-off values across iterations. Variables yielding narrowly distributed thresholds were considered more reproducible.

S8. Outcome variables and rationale for LOS inclusion

Length of hospitalization (LOS) was included as a continuous outcome variable, as it reflects disease severity, clinical evolution, and healthcare resource utilization. Unlike categorical severity classifications, LOS allows the assessment of **linear associations** with biological markers and provides a quantitative measure of disease burden in both MIS-C and acute COVID-19.

The inclusion of LOS enabled complementary analyses alongside severity categories, facilitating evaluation of associations between inflammatory biomarkers, electrolyte disturbances, and clinical course.

S9. Statistical software

All statistical analyses and machine-learning models were implemented using Python (version 3.11.8)**.** Data handling and preprocessing were performed using the *pandas* and *NumPy* libraries. Statistical analyses were conducted with *SciPy* and *statsmodels*. Machine-learning modelling, including penalized regression, Random Forest classification, cross-validation procedures, and performance evaluation, was performed using the *scikit-learn* library.

Visualization of results was carried out using *matplotlib* and *seaborn*. All analyses were executed in a reproducible computational environment.
